# Supplementary material for: Preparation of Hollow Core–Shell Fe3O4/Nitrogen-Doped Carbon Nanocomposites for Lithium-Ion Batteries
Source: Molecules. 2022 Jan 8;27(2):396. doi: 10.3390/molecules27020396 (PMC8781802; doi:10.3390/molecules27020396)
Supplement: Supplementary file 1 [file molecules-27-00396-s001.zip › molecules-1510187-supplementary.pdf]

Supplementary Materials

Preparation of Hollow Core–Shell Fe_3O_4/Nitrogen-Doped Carbon Nanocomposites for Lithium-Ion Batteries

Jie Wang,[1] Qin Hu,[1,2] Wenhui Hu,[1] Wei Zhu,[1] Ying Wei,[1] Kunming Pan,*[3] Mingbo Zheng,*[1,4] and Huan Pang*[1]

[1]School of Chemistry and Chemical Engineering, Yangzhou University, Yangzhou, 225002, Jiangsu, P. R. China

[2] Hengshanqiao senior middle school, Wujin District, Changzhou City, Jiangsu Province

[3]National Joint Engineering Research Center for Abrasion Control and Molding of Metal Materials, & Henan Key Laboratory of High-temperature Structural and Functional Materials, Henan University of Science and Technology, Luoyang 471003, China

[4] College of Materials Science and Technology, Nanjing University of Aeronautics and Astronautics, Nanjing 210016, Jiangsu, P. R. China

* Correspondence: pankunming2008@haust.edu.cn (K.P.); zhengmingbo@nuaa.edu.cn (M.Z.); huanpangchem@hotmail.com (H.P.)

Homepage: https://www.x-mol.com/groups/panghuan

Contents

Experimental Section 3
Characterization. 4
Electrochemical measurements. 5
Preparation of electrode sheet 5
Assembly and performance test of button battery 5
Figure S1. (a) XRD patterns of cubic Fe_2O_3 and standard card; (b) XRD patterns of Fe_3O_4@void@N-Doped C-2 composite and standard card 7
Figure S2. (a, b) SEM images of cubic Fe_2O_3 at different magnification; 8
Figure S3. (a, b) SEM images of cubic Fe_2O_3@PDA at different magnification; 9
Figure S4. (a, b) SEM images of cubic Fe_3O_4@N-Doped C at different magnification; 10
Figure S5. The thermogravimetric analysis curves of Fe_3O_4@void@N-doped C-x composite (x=2, 5 and 10). 11
Figure S6. (a) Cyclic voltammetry curves of the first three cycles of cubic Fe_2O_3; (b) Representative charge-discharge curves of cubic Fe_2O_3 12
Figure S7. (a) Cyclic voltammetry curves of Fe_3O_4@void@N-Doped C-2 composite; (b) Representative charge-discharge curves of Fe_3O_4@void@N-Doped C-2 composite 13
Figure S8. (a) Cyclic voltammetry curves of Fe_3O_4@void@N-Doped C-10 composites; (b) Representative charge-discharge curves of Fe_3O_4@void@N-Doped C-10 composites 14
Figure S9. dQ/dV curves of all samples for C/D profiles during the second cycling 15
Figure S10. 100 cycle diagrams of cubic Fe_2O_3 and Fe_3O_4@void@N-Doped C-x (x =2, 5 and 10) composites at 800 mA g^{-1} current density 16
Figure S11. SEM images of Fe_3O_4@void@N-doped C after 100 cycles. 17
Figure S12. TEM images of Fe3O4@void@N-doped C after 100 cycles. 18
Figure S13. EIS curves of cubic Fe_2O_3 and Fe_3O_4@void@N-Doped C-x composites 19
Figure S14. Field-dependent magnetization curve of Fe_2O_3 measured at room temperature 20

Experimental Section

Chemicals

Iron (III) chloride hexahydrate ($FeCl_3·6H_2O$, 99.99%), sodium hydroxide (NaOH), ammonium hydroxide ($NH_3·H_2O$, 25-28%), 3-hydroxytyramine hydrochloride ($C_8H_{12}ClNO_2$), tris buffer solution (1.0 mol L^{-1} pH = 8.5), hydrochloric acid (HCl, 36-38%), N-methylpyrrolidone (C_5H_9NO), ethanol were purchased from Shanghai Sinopharm Chemical Reagent Co, Ltd. (Shanghai, China). All chemicals were utilized as received without further purification.

Synthesis of Fe_2O_3 cubes. First, take 50 mL of the prepared 2 mol L^{-1} $FeCl_3$ solution into a 150 mL beaker, and stir it in a constant temperature water bath at 75 ºC. Then slowly add 50 mL 5.4 mol L^{-1}NaOH solution and stir it until the brick-red colloid appears. Continue stirring for another 5 minutes, transfer the colloid to a reactor with a volume of 150 ml, and then conduct hydrothermal reaction at 100 °C, The reaction time was 96 hours. The final product was collected by centrifugation and washed by deionized water and ethanol for 2-3 times respectively. The obtained sample is cubic Fe_2O_3, which is brick red powder.

Synthesis of Fe_2O_3@PDA. Take 80 mg cubic Fe_2O_3 and disperse it in 100 mL tris buffer solution (1.0 mol L^{-1}, pH = 8.5). After ultrasonic for 30 minutes, stir it on a magnetic stirrer, add 40 mg 3-hydroxytyramine hydrochloride, and then continue stirring for 6 hours. The final product was collected by centrifugation and washed by deionized water and ethanol for 2-3 times respectively. The obtained sample is Fe_2O_3@PDA (PDA is polydopamine) cube.

Synthesis of cubic massive hollow core-shell Fe_3O_4/N-doped carbon composites. Fe_2O_3@PDA is heat-treated at 700 °C for 3 hours (heating rate 5 °C min^{-1}) in nitrogen atmosphere to obtain carbon-coated sample Fe_3O_4@N-Doped C, which is black powder. Then etch the product, take 0.2 g of the product and put it into 50 mL of HCl solution with concentration of 2 mol L^{-1} (three groups of samples in total), stir it at 30 °C for 2 hours, 5 hours and 10 hours respectively. The final product was collected by centrifugation and washed by deionized water and ethanol for 2-3 times respectively. The obtained samples are Fe_3O_4@void@N-Doped C cubes with hollow core-shell structures, which are black products. The above three groups of products are named as: Fe_3O_4@void@N-Doped C-2, Fe_3O_4@void@N-Doped C-5 and Fe_3O_4@void@N-Doped C-10.

Characterization.

X-ray diffraction patterns (XRD) obtained from a Bruker D8 Advanced X-ray diffractometer equipped with Cu-Kα radiation (λ=0.15406 nm) using a 2θ range of 5-80 ºC for characterization purposes. Scanning electron microscope (SEM, Zeiss_Supra55) was used for observing the morphology of the samples at an acceleration voltage of 5.0 kV. High-resolution transmission electron microscopy (HRTEM) images, SAED images, and energy dispersive X-ray spectroscopy mapping were captured on a Tecnai G2 F30 transmission electron microscopy at an acceleration voltage of 300 kV. Raman measurement was obtained on Raman Microscopic Imaging Spectrometer (DXRxi). XPS measurement was carried out using an Axis Ultra X-ray

photoelectron spectrometer (Kratos Analytical Ltd., UK) equipped with Al-Kα source (hv = 1486.6 eV).

Electrochemical measurements.

Preparation of electrode sheet

To prepare the working electrode, the as-prepared materials (Fe_3O_4@void@N-Doped C-x (x = 2, 5, and 10) composite material), acetylene black and polyvinylidene fluoride (PVDF) with a mass ratio of 8:1:1 were mixed with N-methyl pyrrolidone (NMP) to form a slurry, and then the slurry was subsequently coated on a copper foil, the mass loading of active material was about 0.53-0.85 mg cm^{-2}, followed by drying in a vacuum oven at 80 °C for 12 h. Finally, electrodes coated with active substances can be obtained. The preparation method of pure α-Fe_2O_3 electrode is the same as that of Fe_3O_4@void@N-Doped C-x composite material.

Assembly and performance test of button battery

The button cell (CR-2032) is assembled in a glove box filled with high-purity argon gas (water pressure < 0.1ppm, oxygen partial pressure < 0.1ppm). The battery are assembled in the following order: a negative shell, a stainless steel washer, a lithium plate, a diaphragm, an electrode coated with an active substance, and a positive shell. An appropriate amount of 1 M $LiPF_6$ electrolyte ($LiPF_6$ dissolved in a 1:1 Diethyl Carbonate(DEC) / ethylene carbonate(EC) solvent) is dripped on both sides of the diaphragm. In order to make the electrolyte fully infiltrate the interior of the electrode material, the newly installed button cell can be tested by standing for more than 10 hours.

Under the condition of room temperature, the LAND battery test system is used to conduct constant current charge and discharge test (including cycle stability and rate

performance) on the installed button battery. The voltage range of charge and discharge is 0.01-3 V. Cyclic voltammetry (CV) and alternating current impedance (EIS) are measured by electrochemical workstation (CHI660E, Shanghai). The scanning speed of CV is 0.5 mV s^{-1}. The frequency test range of the EIS is 0.01-10^5 Hz.

Results and discussion

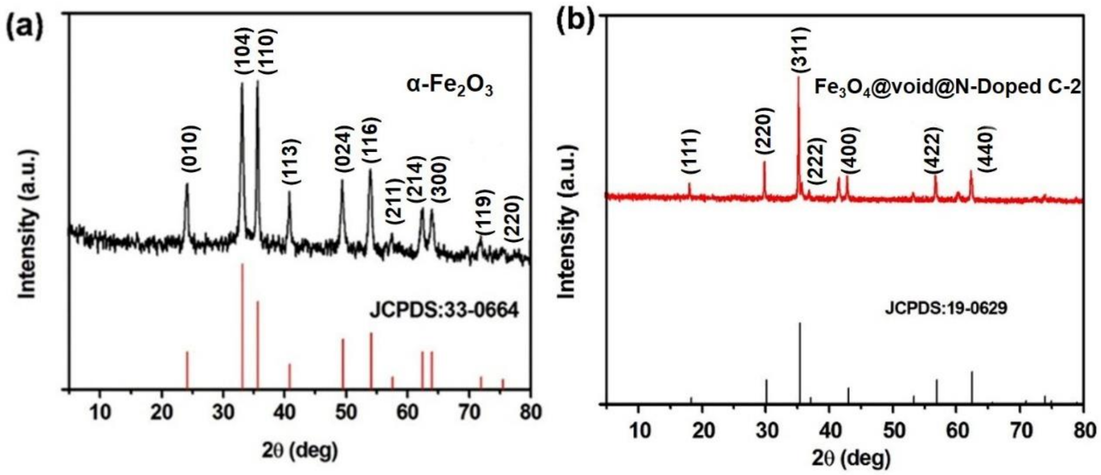

Figure S1. (a) XRD patterns of cubic Fe_2O_3 and standard card; (b) XRD patterns of Fe_3O_4@void@N-Doped C-2 composite and standard card

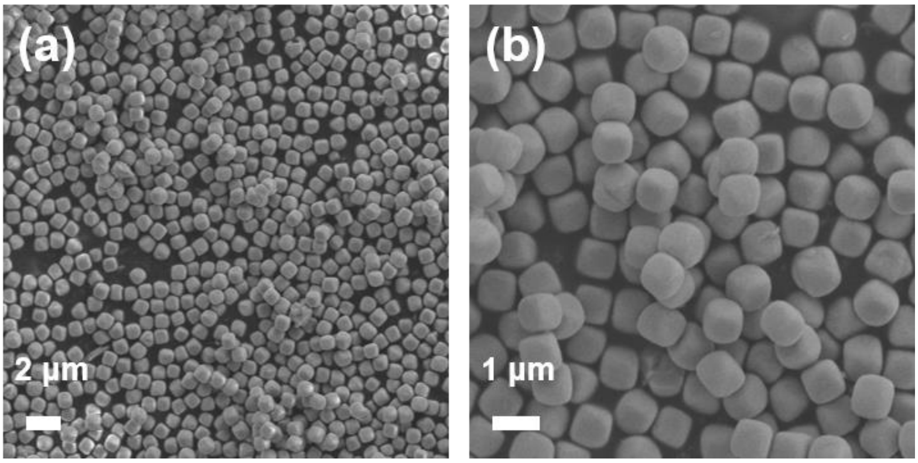

Figure S2. (a, b) SEM images of cubic Fe_2O_3 at different magnification;

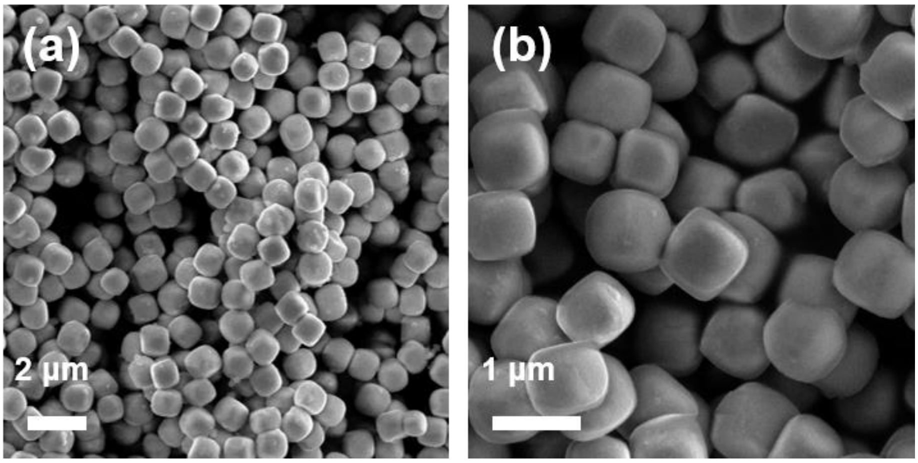

Figure S3. (a, b) SEM images of cubic Fe_2O_3@PDA at different magnification;

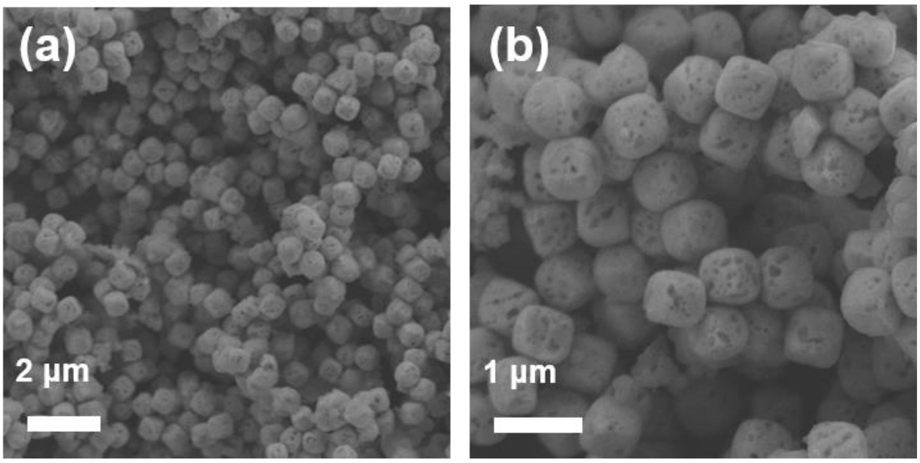

Figure S4. (a, b) SEM images of cubic Fe_3O_4@N-Doped C at different magnification;

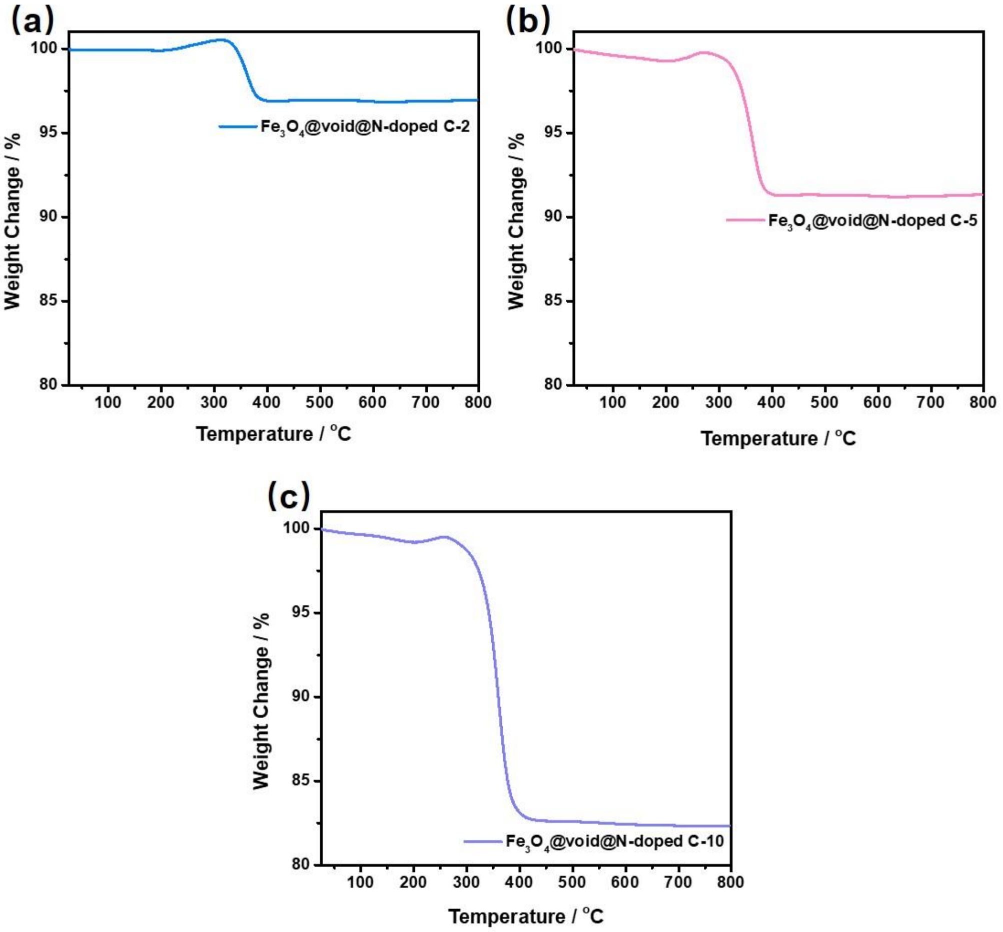

Figure S5. The thermogravimetric analysis curves of Fe_3O_4@void@N-doped C-x composite (x=2, 5 and 10).

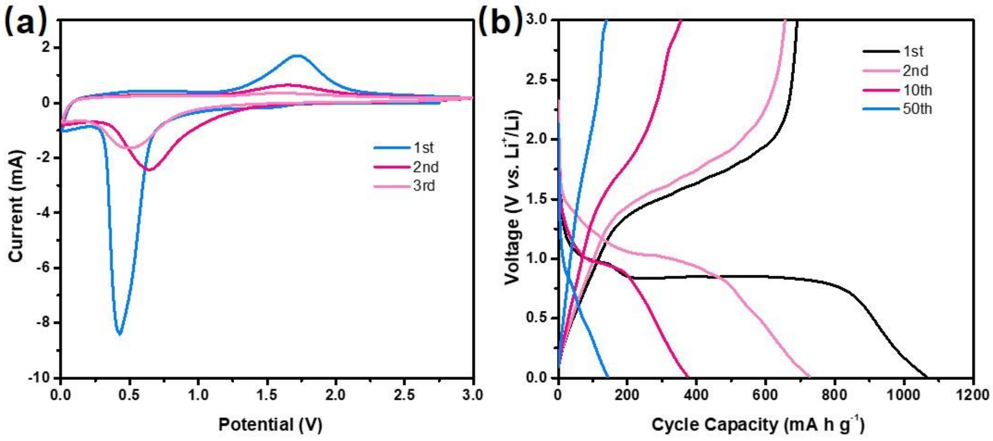

Figure S6. (a) Cyclic voltammetry curves of the first three cycles of cubic Fe_2O_3; (b) Representative charge-discharge curves of cubic Fe_2O_3

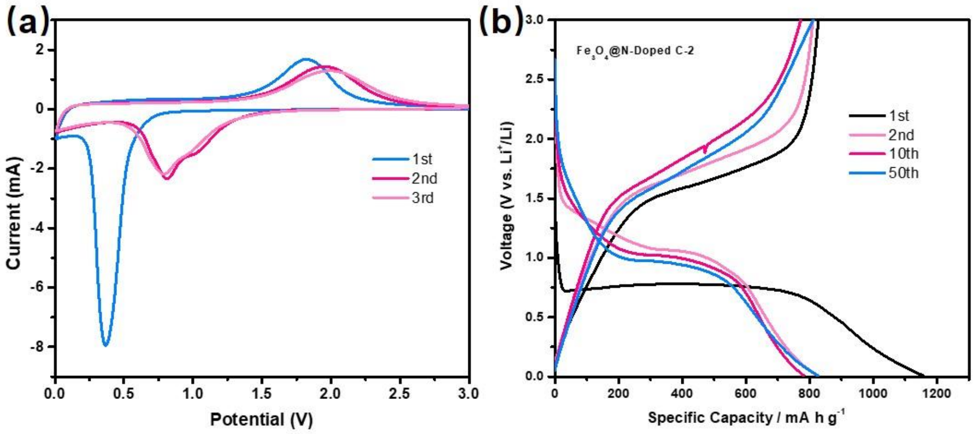

Figure S7. (a) Cyclic voltammetry curves of Fe_3O_4@void@N-Doped C-2 composite; (b) Representative charge-discharge curves of Fe_3O_4@void@N-Doped C-2 composite

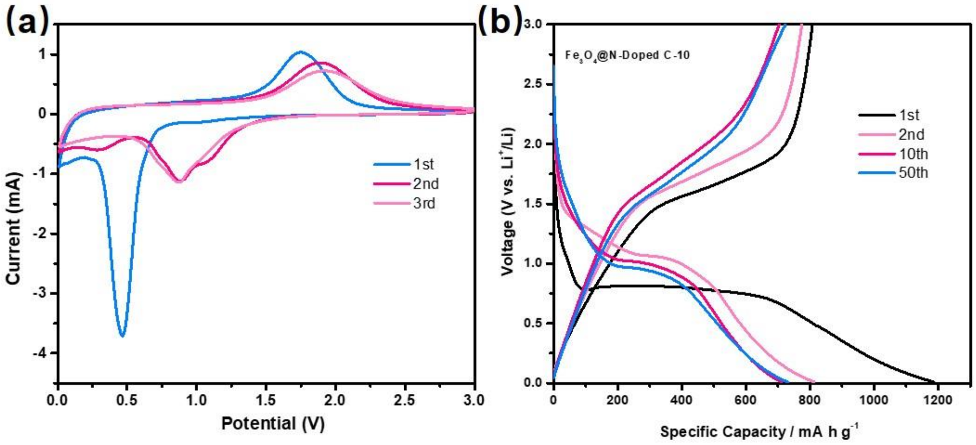

Figure S8. (a) Cyclic voltammetry curves of Fe_3O_4@void@N-Doped C-10 composites; (b) Representative charge-discharge curves of Fe_3O_4@void@N-Doped C-10 composites

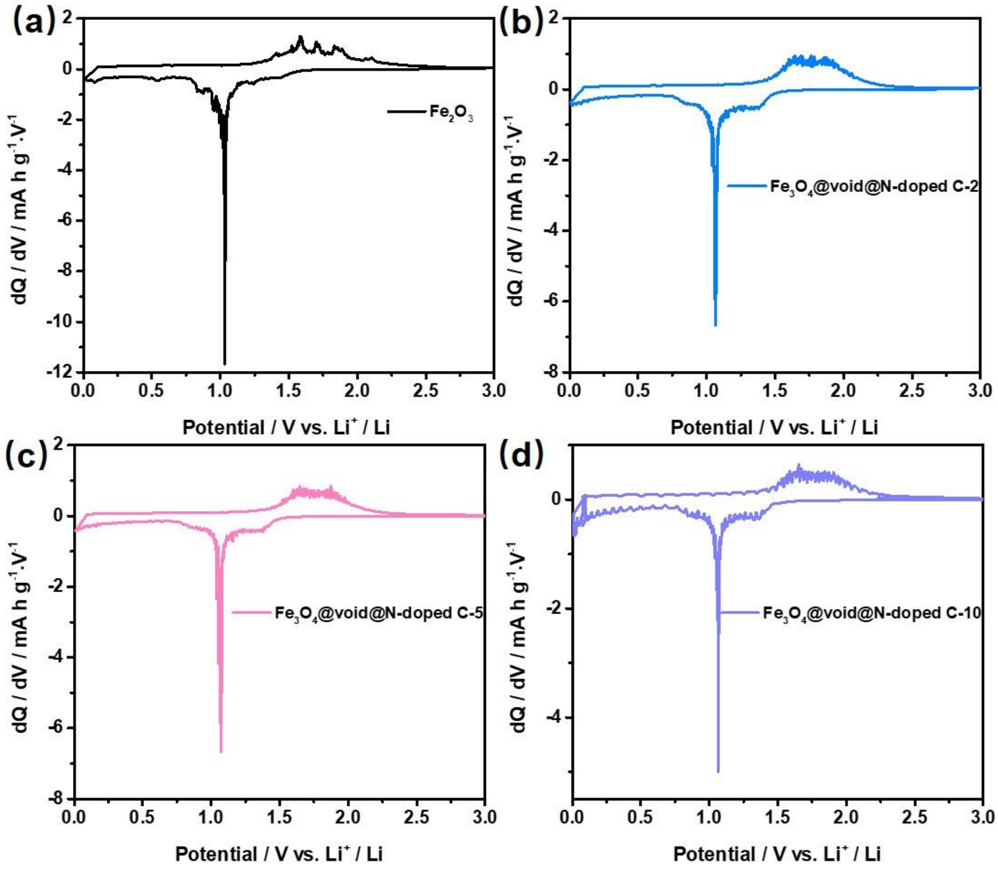

Figure S9. dQ/dV curves of all samples for C/D profiles during the second cycling

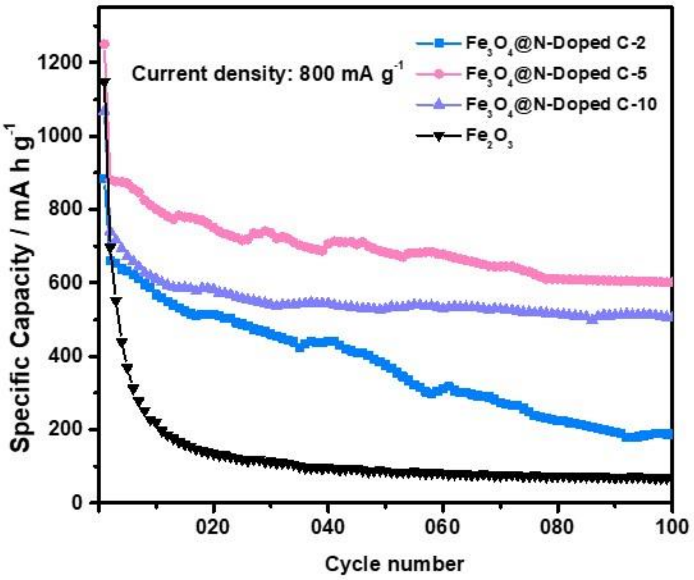

Figure S10. 100 cycle diagrams of cubic Fe_2O_3 and Fe_3O_4@void@N-Doped C-x (x =2, 5 and 10) composites at 800 mA g^{-1} current density

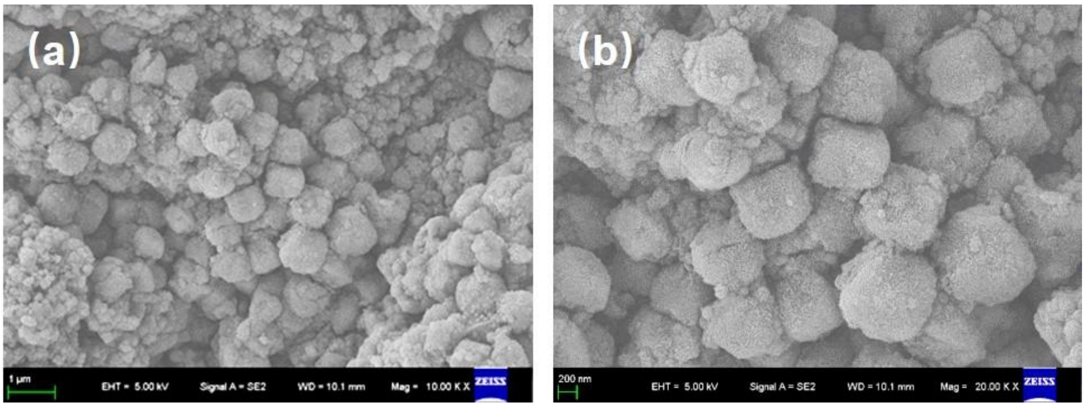

Figure S11. SEM images of Fe_3O_4@void@N-doped C after 100 cycles.

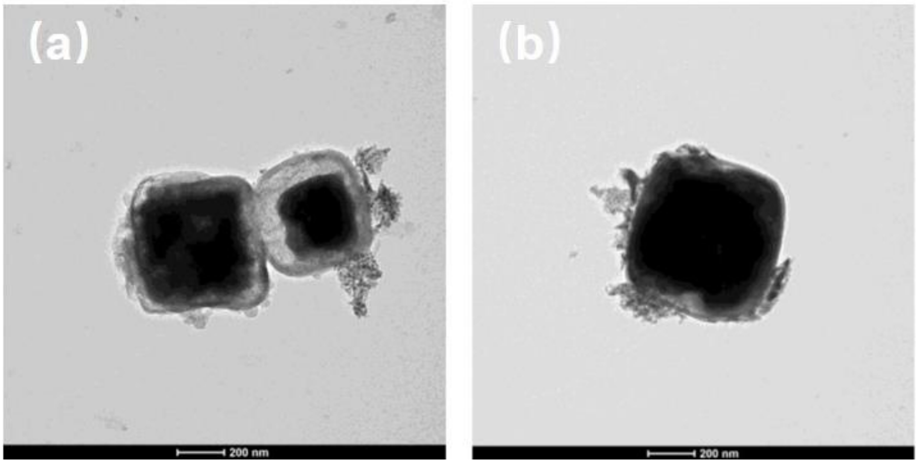

Figure S12. TEM images of Fe3O4@void@N-doped C after 100 cycles.

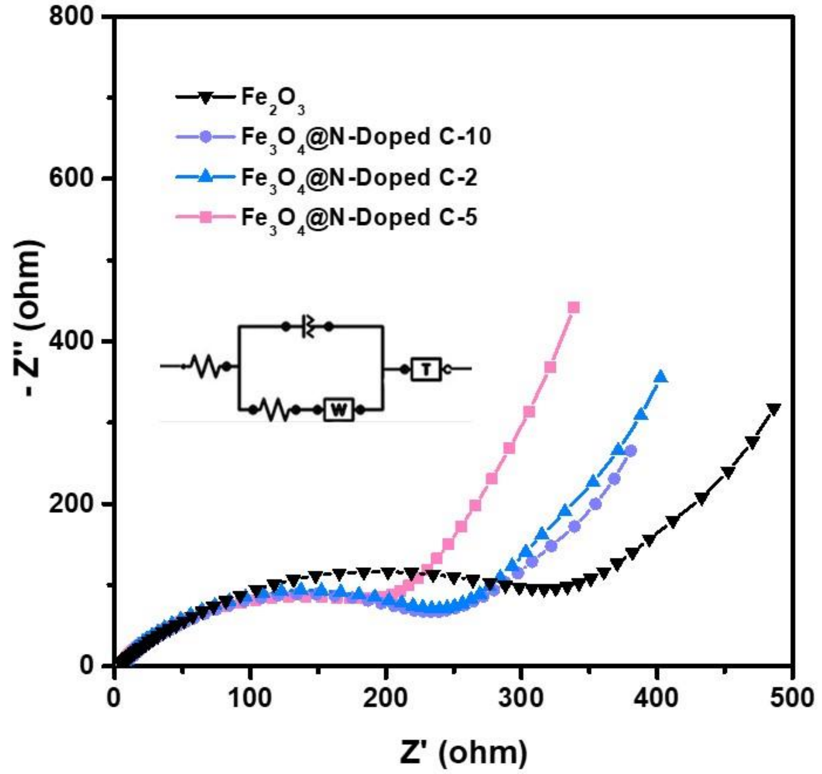

Figure S13. EIS curves of cubic Fe_2O_3 and Fe_3O_4@void@N-Doped C-x composites

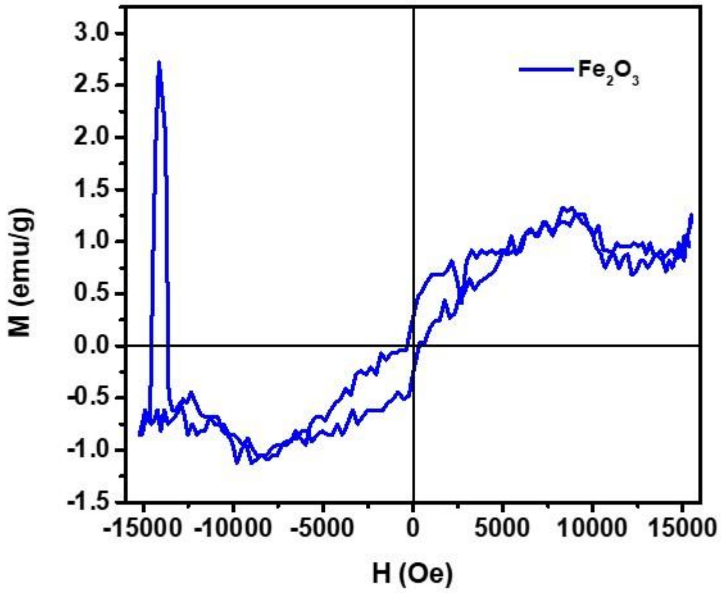

Figure S14. Field-dependent magnetization curve of Fe_2O_3 measured at room temperature
